# Comparative analysis of karyotypes of *Chironomus solitus* Linevich & Erbaeva, 1971 and *Chironomus anthracinus* Zetterstedt, 1860 (Diptera, Chironomidae) from East Siberia

**DOI:** 10.3897/CompCytogen.v9i2.4702

**Published:** 2015-06-03

**Authors:** Valentina Proviz

**Affiliations:** 1 Limnological Institute, SB RAS, Ulan-Batorskaya 3, Irkutsk 664033, Russia

**Keywords:** Karyotype, banding sequences, inversion, *Chironomus solitus*, *Chironomus anthracinus*

## Abstract

A comparative chromosome banding analysis of *Chironomus solitus* Linevich & Erbaeva, 1971 and *Chironomus anthracinus* Zetterstedt, 1860 from East Siberia (Lakes Baikal, Gusinoe, Arakhley and Irkutsk Reservoir) showed close similarity of banding sequences. *Chironomus solitus* differs from *Chironomus anthracinus* in one species-specific sequence of arm B. Arms C (43%) and D (30%) had inversion banding sequences previously reported in *Chironomus anthracinus* The similarity of karyotypic features of *Chironomus solitus* and *Chironomus anthracinus* in combination with morphological features of larvae provide evidence in favour of including *Chironomus solitus* in the *Chironomus anthracinus* group of sibling species long with *Chironomus reservatus* Shobanov, 1997.

## Introduction

*Chironomus solitus* Linevich & Erbaeva, 1971 and *Chironomus anthracinus* Zetterstedt, 1860 are abundant chironomid species (Diptera: Chironomidae), inhabiting the silty bottoms of various water bodies in Pribaikalye and Zabaikalye. *Chironomus solitus* was first registered in the Irkutsk Reservoir as well as in the Angara River, Bratsk, Ust-Ilimsk water reservoirs (Linevich and Erbaeva 1971; Linevich 1981; Proviz et al. 1991; Erbaeva and Safronov 2009), in lakes and rivers of the Barguzin River basin (Buyantuev 1999) and recently encountered in the near-shore zone of Lake Baikal. *Chironomus anthracinus* is a widespread Holarctic species known from the Angara River and its tributaries, Irkutsk Reservoir, lakes of Western Zabaikalye (Linevich and Erbaeva 1971; Linevich 1981; Kiknadze et al. 2005) and the basin of the Barguzin River (Buyantuev 1999).

*Chironomus solitus* and *Chironomus anthracinus* live in the single type water environments (lakes, water reservoirs), and are characterized by similar larval morphology in the features used in the distinction of *Chironomus* species, which makes their differentiation complicated. Thus, accurate identification of these species requires analysis of their karyotypes, rather than only external larval morphology. Until recently, the *Chironomus solitus* karyotype had only been examined in one population from the Irkutsk Reservoir. The first data were reported by Bukhteeva (1979); later, banding chromosome patterns and polymorphisms were described (Proviz 2009). Karyological analysis was made of *Chironomus anthracinus* from many Palearctic and Nearctic regions (Belyanina 1983; Kiknadze et al. 1991, 1996; Shobanov 1996; Petrova and Rakisheva 2003; Kiknadze et al. 2005). In East Siberia, karyotypes of larvae from Lake Shchuchie (Buryatia) were briefly reported by Bukhteeva (1979, 1980). Later, Kiknadze and co-authors (Kiknadze et al. 1991, 1996) described the karyotypic of *Chironomus anthracinus* from the Vilyuy Reservoir (Yakutia).

The present work is aimed at comparative analysis of *Chironomus solitus* and *Chironomus anthracinus* karyotypes from the largest lakes of East Siberia, Baikal, Gusinoe, Arakhley and Irkutsk Reservoir, and determination of cytogenetic features for their identification.

## Material and methods

Fourth instar larvae of *Chironomus solitus* were collected in January 1992 in the Irkutsk Reservoir (depth 3 m, 52 larvae), and in June 2008 in Lake Baikal opposite the Bolshye Koty Settlement (depth 6 m, 12 larvae). *Chironomus anthracinus* were collected in May 2013 in Lake Gusinoe (10–22 m, 65 larvae), and in March 2014 in Lake Arakhley (10–17 m, 78 larvae). Larvae were fixed in a 3:1 mixture of 96% ethanol and glacial acetic acid. Karyological preparations were made using the ethyl-orcein method (Demin and Shobanov 1990). In 1992 and 2008, chromosomes were photographed by a micro-camera unit MCU-1 with 90× zoom magnification; in 2013–2014, this was performed using an Axiostar plus (Zeiss) microscope (Centre for Microscopic Analysis LIN SB RAS) with AxioVision Rel. 4.7.1 software. Mapping of arms A, C, D, E, and F of *Chironomus anthracinus* chromosomes was performed according to Kiknadze et al. (2005) based on piger-standard (Keyl 1962, Devai et al. 1989), while standard map of *Chironomus plumosus* suggested by Shobanov (1994, 1996) was used for mapping of arm B. Symbols designating banding sequences are as follow: distribution areas marked by p’ for Palearctic, n’ for Nearctic, and h’ for Holarctic zoogeographical regions (Kiknadze et al. 2005) and followed by abbreviated species name (sol), arm designation (A) and banding sequence number–p’solx1 (in homozygote–p’solA1.1).

## Results

### Larval morphology

Both species have a light yellow (from the dorsal part) cephalic capsule, including the frontal sclerite. Abdominal segment VIII bears two pairs of long ventral appendages; lateral appendages on segment VII are absent (bathophilus type after: Lenz 1926). Premandible with two uneven teeth. Fourth lateral cusp of mentum is smaller than fifth cusp. Third antennal segment is shorter than the fourth. The colour of the fourth lower mandibular tooth varies; that of *Chironomus solitus* is dark yellow, while the remaining teeth are dark brown. The results of our examination of the population from Lake Arakhley showed that *Chironomus anthracinus* tooth was either dark yellow or of the same colour as the rest of the teeth.

### Karyotype characteristics

**Figure 1.**
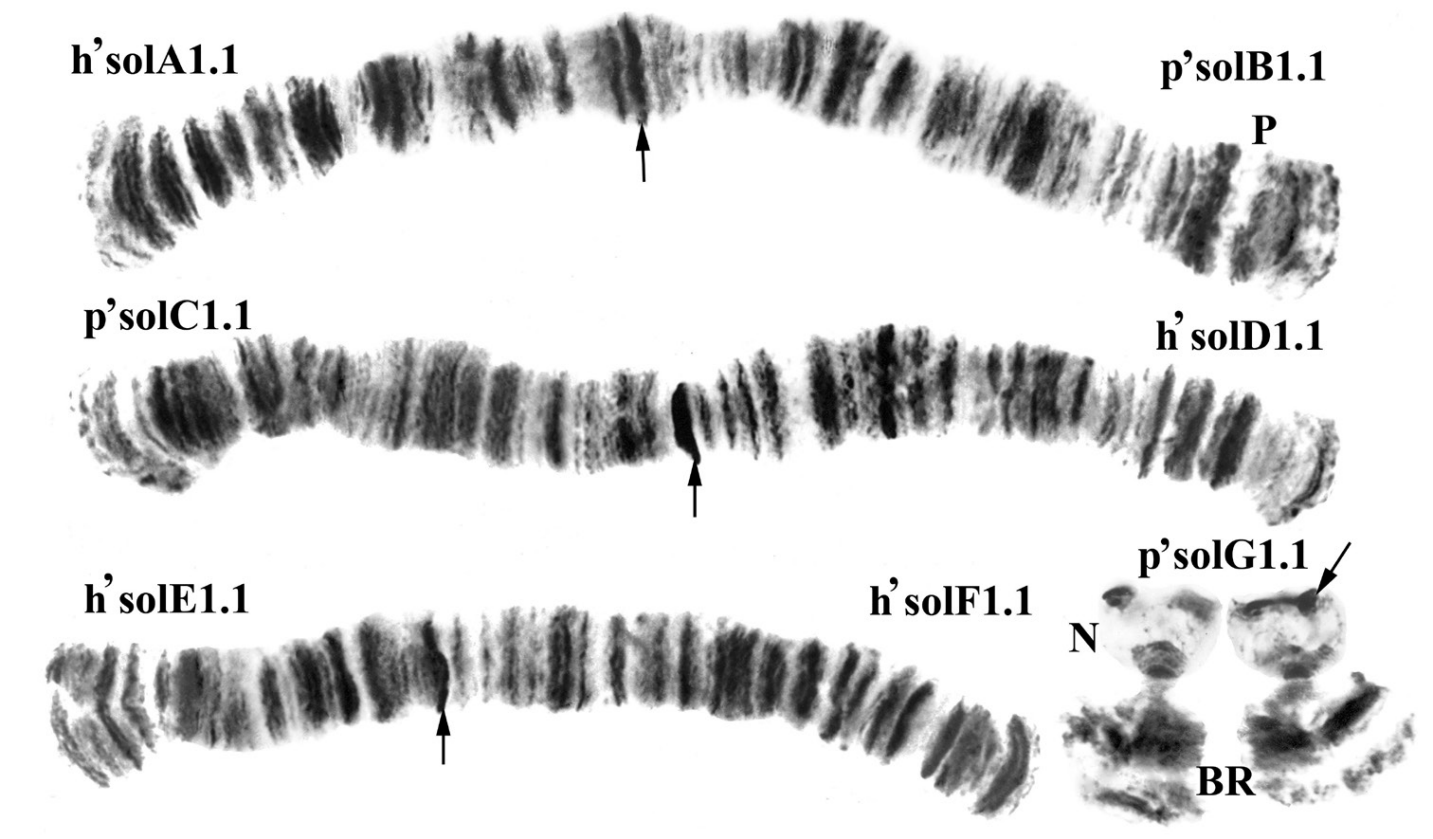
Karyotype of *Chironomus solitus*. h’solA1.1, p’solB1.1 ets.–genotypic combinations of banding sequences in chromosomal arms; N – nucleolus; BR – Balbiani Ring, p – puff, arrows show centromeric bands.

**Figure 2.**
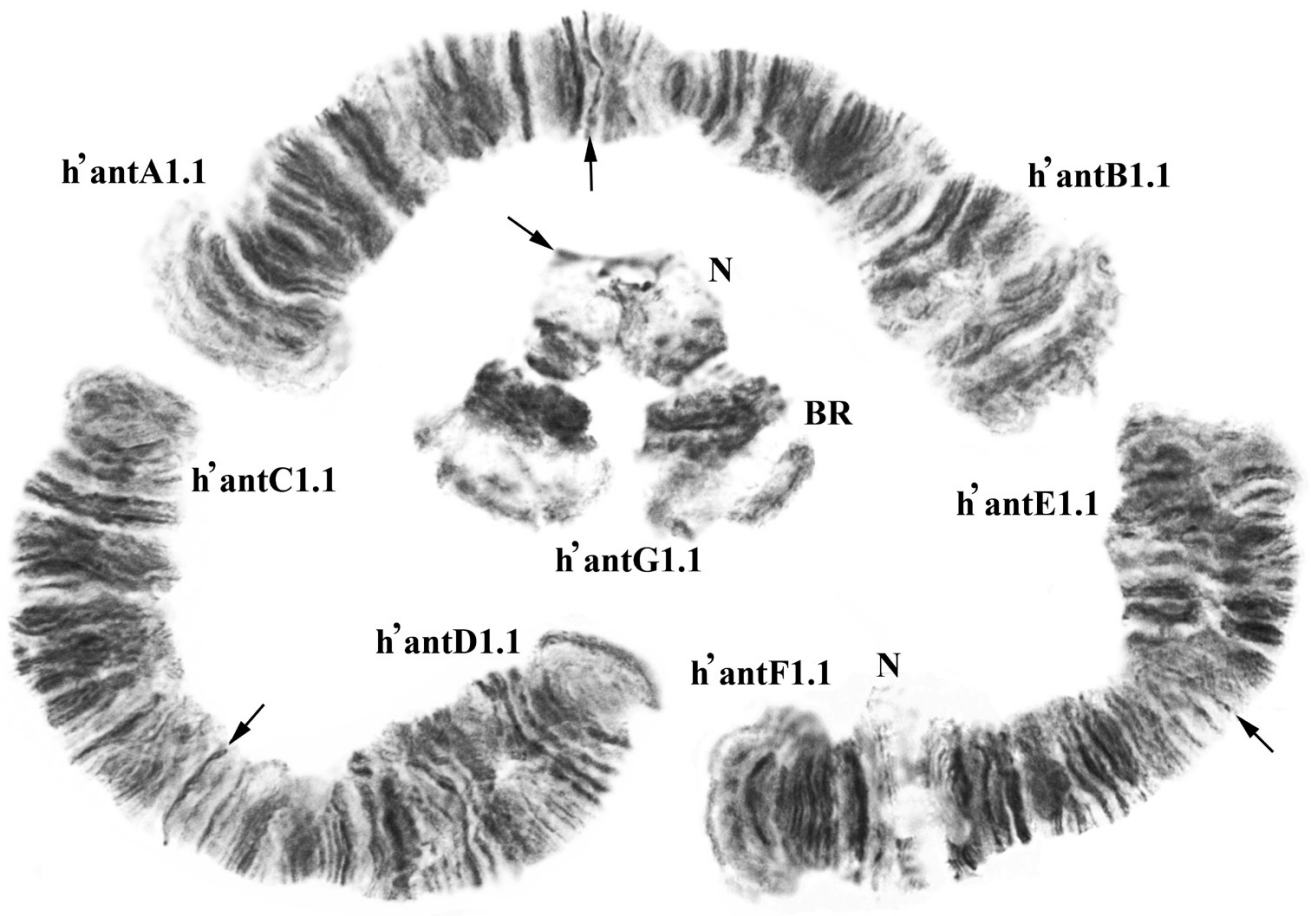
Karyotype of *Chironomus anthracinus*. The designations are the same as in Fig. 1.

Karyotypes of *Chironomus solitus* (Fig. 1) and *Chironomus anthracinus* (Fig. 2) have common morphological features: 2n=8. A combination of chromosome arms is typical for species from “thummi” cytocomplex. Chromosomes AB and CD are metacentric, EF, submetacentric, and G, telocentric. The species differ in the size of cenromeric heterochromatin. The centromeric areas of *Chironomus solitus* are well defined, and the centromeres of *Chironomus anthracinus* look like thin disks. Arm G homologues are unconjugated and carry a Balbiani Ring (BR) and a nucleolus (N). In *Chironomus anthracinus* there is a second nucleolus in the arm F.

### Banding sequences

**Figure 3.**
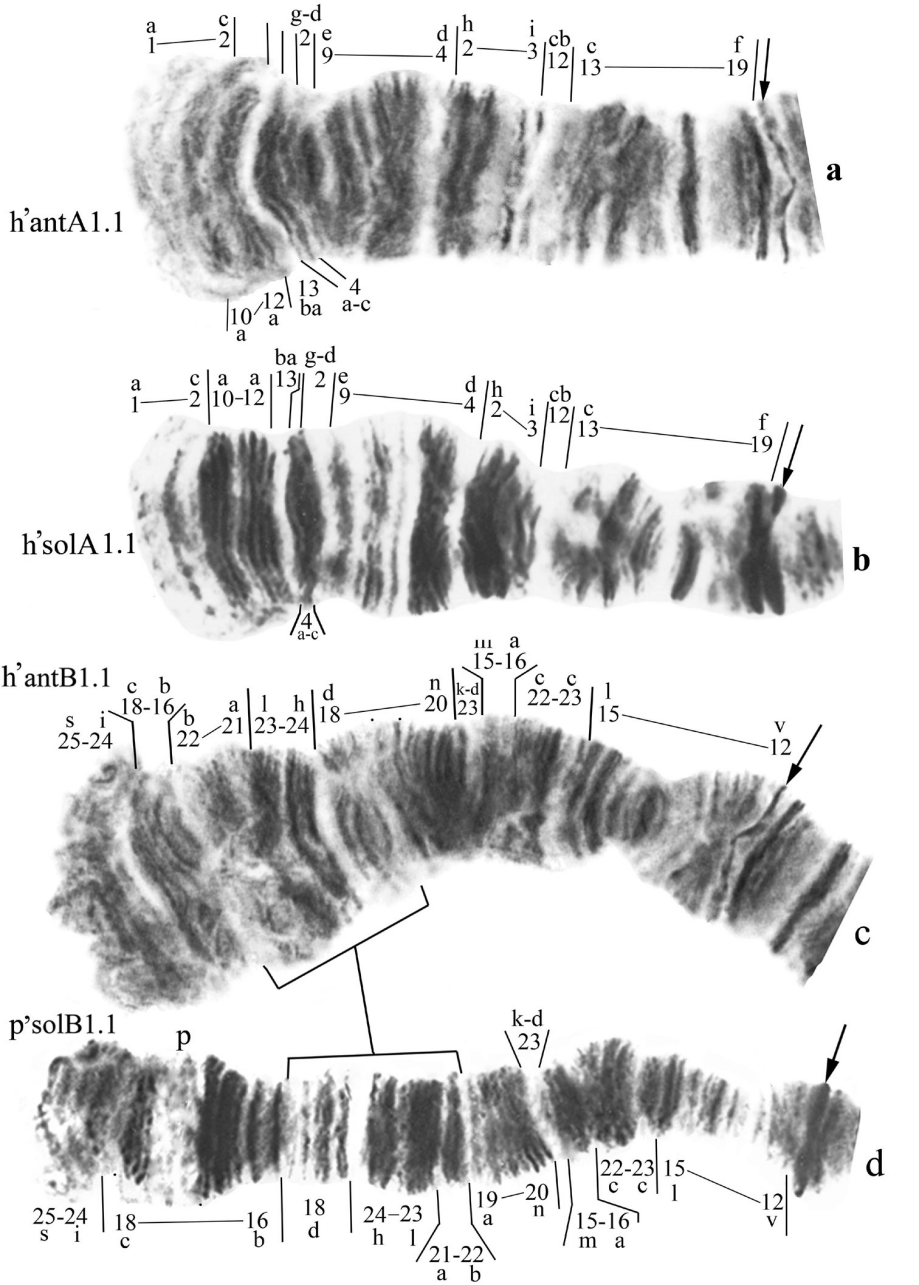
Homozygous banding sequences in the arms A and B of *Chironomus anthracinus* and *Chironomus solitus*. **a** h’antA1.1 **b** h’solA1.1 **c** h’antB1.1 **d** p’solB1.1. Numbers and small letters under chromosome arm correspond to banding sequences, brackets near chromosome arms show inversions.

**Arms A** of *Chironomus anthracinus* and *Chironomus solitus* are monomorphic with a single identical banding sequence h’antA1=h’solA1 (Fig. 3, a, b): h’antA1= h’solA1 1a-2c 10a-12a 13ba 4a-c 2g-d 9e-4d 2h-3i 12cb 13c-19f C

**Arms B** of *Chironomus anthracinus* and *Chironomus solitus* are monomorphic with banding sequences h’antB1 (Fig. 3, c) and p’solB1 (Fig. 3, d) differ by a simple inversion:

h’antB1 25s-24i 18c-16b 22b-21a 23l-24h 18d-20n 23k-d 15m-16a 22c-23c 15l-12v C

p’solB1 25s-24i 18c-16b 18d 24h-23l 21a-22b 19a-20n 23k-d 15m-16a 22c-23c 15l-12v C

In addition to the inversion, *Chironomus solitus* differs from *Chironomus anthracinus* by the presence of a puff in the region 17. The banding sequence h’antB2 (Kiknadze et al. 2005) were found in Palearctic and Nearctic *Chironomus anthracinus* populations. The borders of this inversion located close to the borders of inversion that differ banding sequence p’solB1 from h’antB1. Standard mapping of *Chironomus plumosus* (Shobanov, 1994) allows it to be represented as follows:

h’antB2 25s-24i 18c-17a 23l 21a-22b 16b 24h 18d 19a-20n 23k-d 15m-16a 22c-23c 15l-12v C

**Figure 4.**
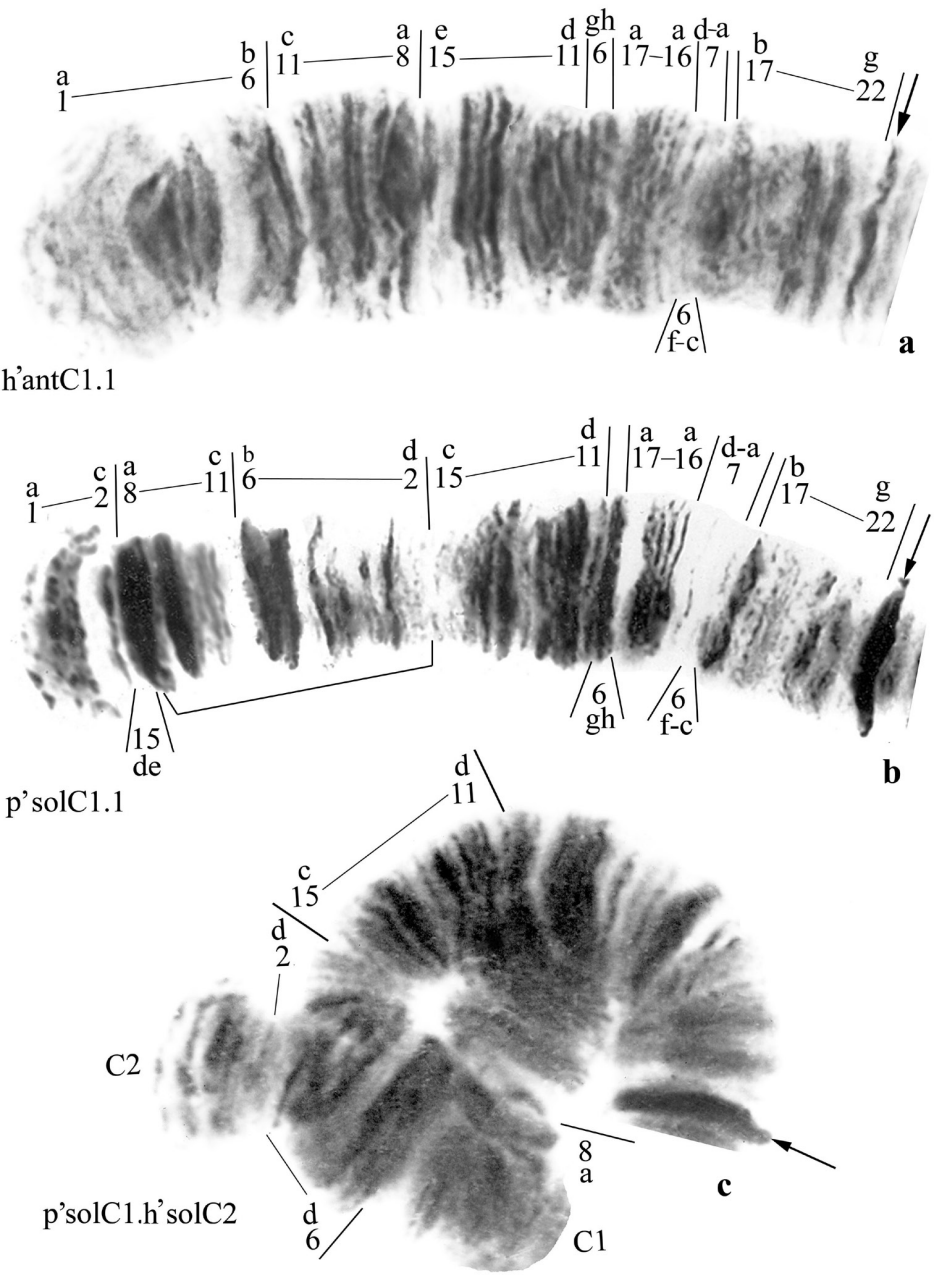
Banding sequences in the arm C of *Chironomus anthracinus* and *Chironomus solitus*. **a** homozygotes h’antC1.1. **b** homozygotes p’solC1.1 **c** heterozygous inversions p’solC1.h’solC2. Designations as in Fig. 3.

**Arm C** of *Chironomus anthracinus* is monomorphic, with a single banding sequence h’antC1 (Fig. 4, a). Arm C of *Chironomus solitus* is polymorphic and has two banding sequences–p’solC1 (Fig. 4, b) and h’solC2–differing by one simple inversion (Fig. 4, c). Inversion heterozygotes p’solC1.h’solC2 made up 25% and 43% of Baikal and Irkutsk Reservoir populations, respectively. The same banding sequences, h’antC1 (=h’solC2) and p’antC2 (=p’solC1), were also registered in *Chironomus anthracinus* populations from other localities within this area, although in somewhat different proportions:

h’antC1= h’solC2 1a-2c 2d-6b 11c-8a 15ed 15c-11d 6gh 17a-16a 7d-a 6f-c 17b-22g C

p’solC1=p’antC2 1a-2C 15de 8a-11c 6b-2d 15c-11d 6gh 17a-16a 7d-a 6f-c 17b-22g C

h’antC1 sequence dominated in all of the populations studied, while p’antC2 was less common and occurred in both homo- and heterozygous states (Kiknadze et al. 2005).

**Figure 5.**
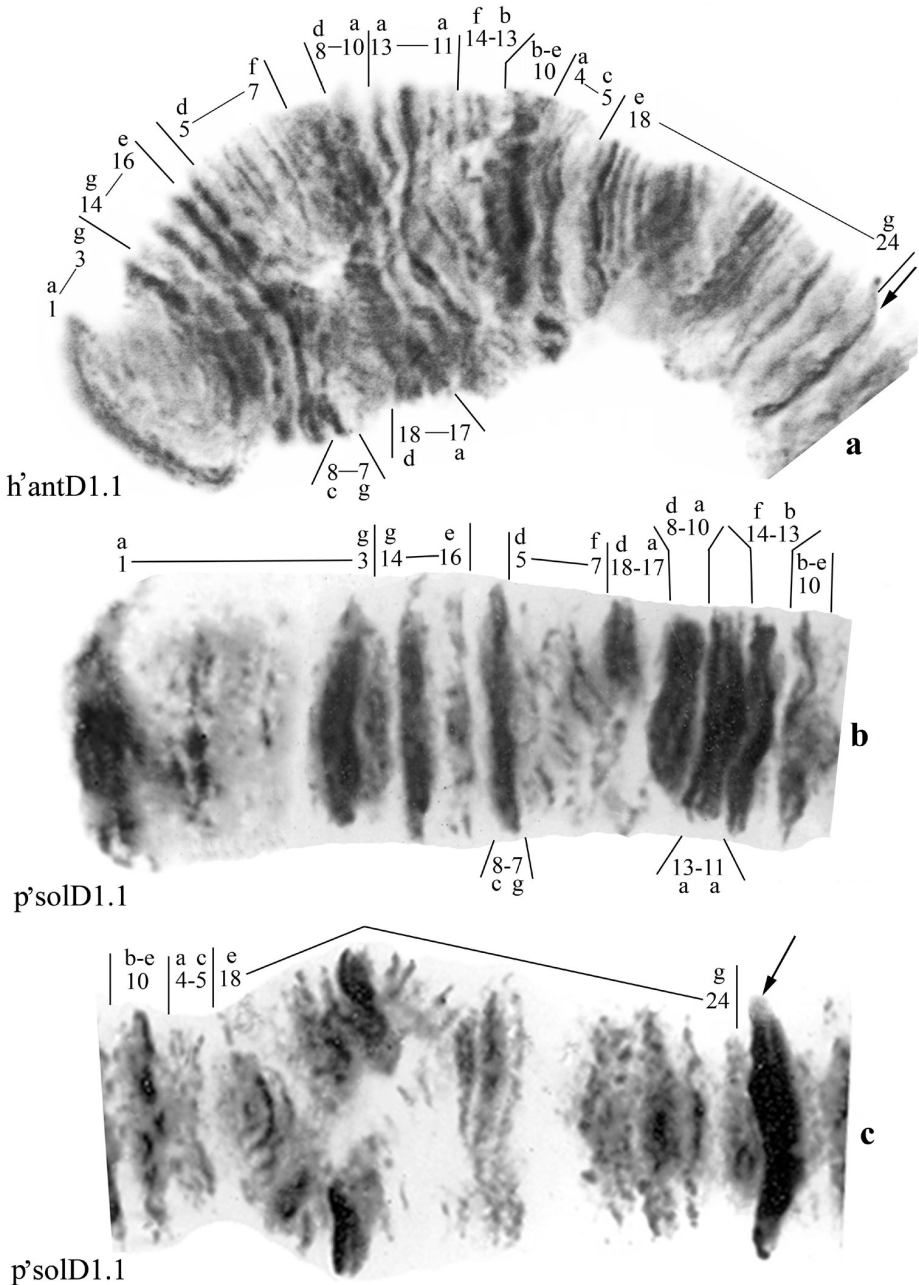
Homozygous banding sequences in the arm D of *Chironomus anthracinus* and *Chironomus solitus*. **a** h’antD1.1 **b** and **c** h’solD1.1. Designations as in Fig. 3.

**Figure 6.**
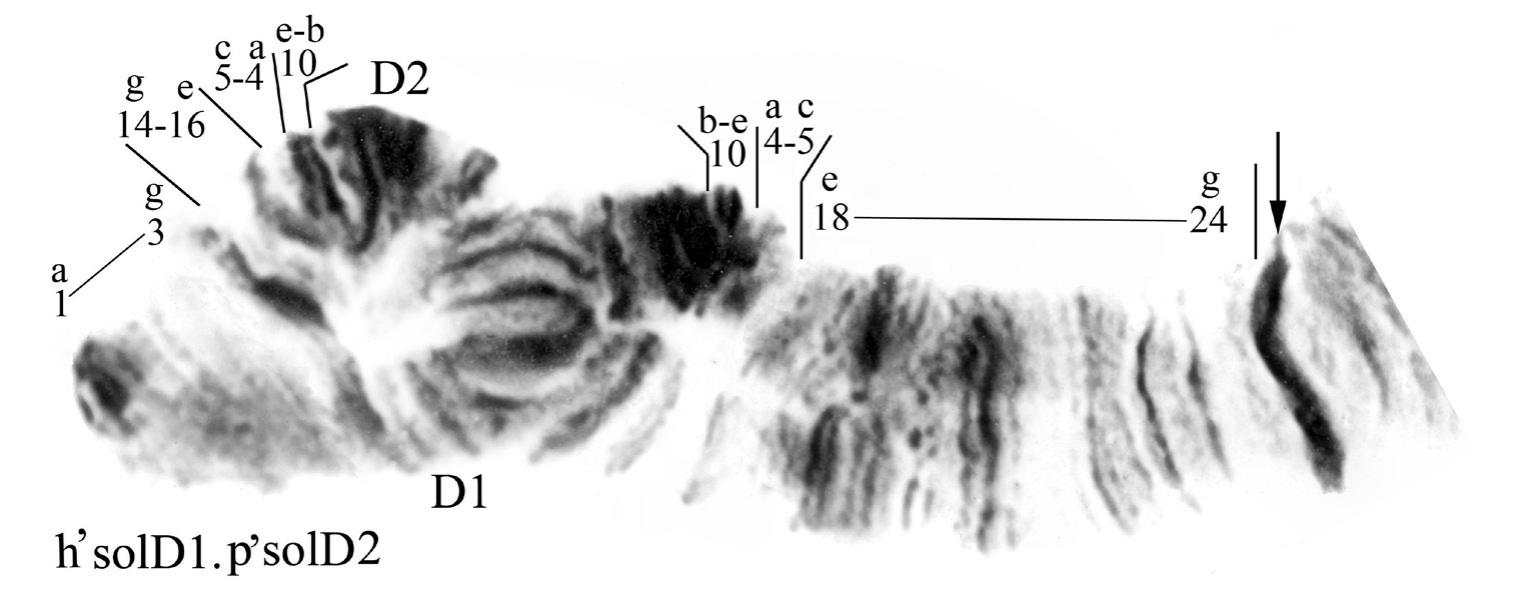
Inversion heterozygote h’solD1.p’solD2 in the arm D *Chironomus solitus*. Designations as in Fig. 3.

**Arm D** of *Chironomus anthracinus* is monomorphic, with one h’antD1 banding sequence (Fig. 5, a). Arm D of *Chironomus solitus* is polymorphic and has two banding sequences–h’solD1 (Fig. 5, b, c), identical to h’antD1, and p’solD2 (Fig. 6), which differs by a simple inversion. Inversion heterozygotes h’solD1.p’solD2 were found in 17% of specimens from the Baikal population, and in 30% from the Irkutsk Reservoir. *Chironomus anthracinus* from western parts of Palearctics also had a p’antD2 banding sequence identical to that of p’solD2 and was found in homo- and heterozygous states (Kiknadze et al. 2005):

h’antD1=h’solD1 1a-3g 14g-16e 8c-7g 5d-7f 18d-17a 8d-10a 13a-11a 14f-13b 10b-e 4a-5c 18e-24g C

p’antD2=p’solD2 1a-3g 14g-16e 5c-4a 10e-b 13b-14f 11a-13a 10a-8d 17a-18d 7f-5d 7g-8c 18e-24g C

**Figure 7.**
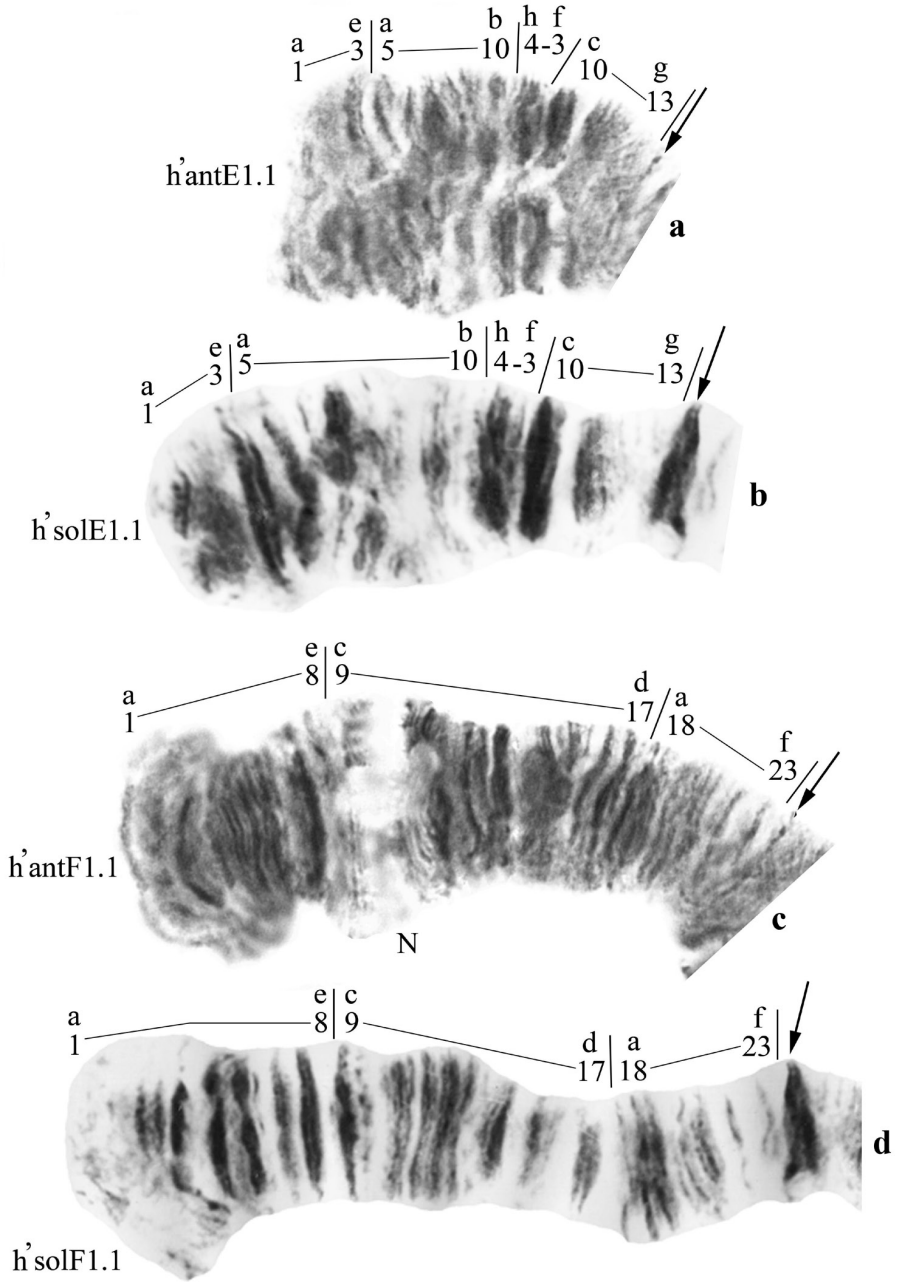
Homozygous banding sequences in the arms E and F of *Chironomus anthracinus* and *Chironomus solitus*. **a** h’ant E1.1 **b** h’solE1.1 **c** h’antF1.1 **d** h’solF1.1.

**Arms E** of *Chironomus anthracinus* (Fig. 7, a) and *Chironomus solitus* (Fig. 7, b) are monomorphic and have an identical banding sequence:

h’antE1= h’solE1 1a-3e 5a-10b 4h-3f 10c-13g C

**Arms F** of *Chironomus anthracinus* (Fig. 7, c) and *Chironomus solitus* (Fig. 7, d) also have an identical banding sequence that is only found in a homozygous state in East Siberia:

h’antF1= h’solF1 1a-8e 9c-17d 18a-23f C

A second nucleolus in Arm F of *Chironomus anthracinus* is a species-specific feature of *Chironomus anthracinus* that makes it different from *Chironomus solitus* with a single nucleolus in arm G.

**Arms G** of *Chironomus anthracinus* and *Chironomus solitus* (Figs 1, 2) have similar morphology: unconjugated homologues with a constriction, unclear banding pattern, similar location of Balbiani Ring and nucleolus. In general, homologues have ectopic contacts in active loci.

## Discussion

As a result of comparative analysis of banding patterns of *Chironomus solitus* and *Chironomus anthracinus* from East Siberia, the similarity of these species in morphological features of larvae as well as karyotypes was revealed. Most of the chromosomal arms, A, D, E and F, have identical banding sequences, and a similar structure of arm G. The principal distinctive features of *Chironomus solitus* karyotype are the species-specific p’solB1 sequence and the absence of a nucleolus in arm F. Previous investigators (Belyanina 1979, Kiknadze et al. 1991, 1996, Shobanov 1996, Petrova and Rakisheva 2003, Kiknadze et al. 2005) reported a low level of chromosome polymorphisms in *Chironomus anthracinus*. Analysis of the populations with standard banding sequences from Lakes Gusinoe and Arakhley also confirmed these observations. The overall banding sequence pool of *Chironomus anthracinus* from other regions includes h’antC2, h’antC1 and p’antD2 sequences, which are identical to p’solC1, h’solC2 and p’solD2 from East Siberia; this is suggestive of karyological similarity of *Chironomus solitus* and *Chironomus anthracinus*.

There is one more species of the genus *Chironomus* – *Chironomus reservatus* Shobanov, 1997, which has close similarity of karyotypic and morphological features at all developmental instars of *Chironomus anthracinus* (Shobanov, 1997). Based on these results, the author included the two species in the *Chironomus anthracinus* group. Banding sequence p’resB1, alongside h’antB2, localised close to p’solB1, and is regarded one of the species-specific markers:

p’resB1 25s-24i 18c-16b 22b-18d 24h-23d 15m-16a 22c-23c 15l-12v C

The morphology of *Chironomus anthracinus* and *Chironomus solitus* imagines from East Siberia is insufficiently studied (Linevich and Erbaeva 1971), therefore, it is possible to compare only several characteristics of these species. For instance, AR of *Chironomus solitus* (3.8) is most closely related to *Chironomus anthracinus* (4.14–4.43) from the European part (Shobanov 1996), and *Chironomus anthracinus* from East Siberia (5.0) – to *Chironomus reservatus* (4.8–5.6) (Shobanov 1997). Further research into metamorphosis of these species should be conducted to make reliable conclusions.

The results of our investigation, similarity of karyotypic features of *Chironomus solitus* and *Chironomus anthracinus* in combination with morphological features of larvae provide evidence in favour of their close similarity and enable us to include *Chironomus solitus* as well as *Chironomus reservatus* in the *Chironomus anthracinus* group.
